# Comparative outcomes of central versus lateral lip-split access in head and neck reconstructive surgery following tumor resection

**DOI:** 10.6026/973206300221346

**Published:** 2026-03-31

**Authors:** Gunjan Virendra Manchanda, Dhrubajyoti Deka, Praveen Kumar, Sunil Kumar Gulia, Ritoban Saha Bhowmick, K Tejaswini, Rahul Tiwari

**Affiliations:** 1 Department of Otorhinolaryngology, Government Medical College and Maharashtra Post Graduate Institute of Medical Education and Research (MUHS), Nashik, Maharashtra, India; 2 Department of Plastic Surgery, Assam Medical College and Hospital, Dibrugarh, Assam, India; 3 Department of Oral and Maxillofacial Surgery, Vanachal Dental College and Hospital, Jharkhand, India; 4 Department of Oral and Maxillofacial Surgery, SGT University, Gurugram, Badli, Jhajjar, Haryana, India; 5 Department of Oral and Maxillofacial Surgery, Manipal Hospitals, Dhakuria, Kolkata, West Bengal, India; 6 Consultant, Oral and Maxillofacial Surgeon, Bellary, Karnataka, India; 7 Department of Oral and Maxillofacial Surgery, RKDF Dental College and Research Centre, Sarvepalli Radhakrishnan University, Bhopal, Madhya Pradesh, India

**Keywords:** Mandibulotomy, lip-split, head and neck reconstruct, micro vascular, free flap

## Abstract

Optimal incision design for lip-split mandibulotomy in advanced head and neck tumor resection remains controversial, as postoperative functional impairment and aesthetic morbidity continue to affect long-term quality-of-life despite adequate oncologic clearance. Therefore, it is of interest to compare lateral and central access via the lips of patients undergoing multi-tumoral resection as well as microvascular reconstruct. The lateral lip-split approach showed less injuries and better postoperative oral performance, whereas central lip-split access was slightly shorter exposure times to the operation. Both options are safe but lateral lip-split access has superior aesthetic and functional results. The choice of an incision's design must consider oncologic access as well as the long-term quality of life outcomes.

## Background:

Advanced stage cancers of the oral cavity and oropharynx often necessitate jaw-splitting excision (composite resection) for adequate oncologic clearance, with subsequent microvascular reconstruction of composite defects. Lip split mandibulotomy continues to be one of the classical surgical access procedures as it offers a direct visualization of the deep tumor margins and recipient vessels [1]. However, postoperative morbidity such as lip scar, oral incompetence [2], salivary leakage and speech disorder still remain an issue. Variations of lip-splitting incisions, such as central versus lateral approaches, are suggested to achieve a more satisfactory cosmetic and functional result while maintaining the same level of exposure [3]. Quality-of-life endpoints deserve increasing emphasis in contemporary reconstructive practice with oncologic safety [4]. Comparative clinical data on the different incisions are still scarce, especially in the context of free-flap reconstruction and fast recovery programs [5]. Therefore, it is of interest to investigate central lip-split and lateral lip-split approaches for their feasibility, complication rates and functional reconstruction in patients who underwent a tumor resection/ reconstruction.

## Materials and Methods:

Prospective comparative cohort study at a tertiary head and neck oncology centre. They consisted of adult patients (18-70 years) undergoing oral or oropharyngeal squamous cell carcinoma lateral mandibular resection requiring a mandibulotomy and free flap reconstruction.

## Exclusion criteria:

Past lip surgery, recurrent tumors and any systemic disease. Based on types of incisions the subjects were grouped as: Group A comprised of central lip-split mandibulotomy and Group B contained lateral split lip incision without damaging the philtral column. The oncologically adequate margins were maintained for all resections and radial forearm or fibula free flaps were used in reconstruction. Operative exposure time, wound complications, lip incompetence and scar satisfaction were the main outcomes. Flap survivals, length of stay and speech intelligibility were the secondary outcomes. Lip competence was tested using a validated Oral Incompetence Score and scar aesthetics was tested using the Patient and Observer Scar Assessment Scale. The follow-up was done at 1, 3 and 6 months. The statistical procedures were independent t-test on continuous variable and Chi-square test on categorical variable with level of significant value being p<0.05.

## Results:

Central lip-split reflected a much lower time of exposure to operation when we compare it to the lateral lip-split position (18.6 ± 3.2 vs 20.1 ± 3.5 minutes; p = 0.04). Nonetheless, the central incision group had significantly higher wound-related complications. Similar to the wound dehiscence (18.7% vs 5.8% p = 0.03) and salivary leakage (15.6% vs 5.8% p = 0.04). The success rates of the free-flaps were also comparable between the two groups (96.9% vs 97.1; p = 0.92). The lateral lip-split technique also reduced the length of stay (10.8 ± 1.9 vs 12.4 ± 2.1 days; p = 0.02) demonstrating that the technique led to a faster postoperative recovery and lower wound morbidity (Table 1 - see PDF). Access to the tongue with lateral lip splits demonstrated greater functional recovery. Speech intelligibility and oral score were higher for the group with lateral access. Patients also reported better satisfaction with scars and overall quality of life scores. These results indicate that incisions with lateral lip splits are more effective to protect the perioral muscles and lessen the risk of contracture-related problems (Table 2 - see PDF).

## Discussion:

Lip-split mandibulotomy is required in wide excision in complex head and neck excisions. Classical bilateral lip-splitting central incisions are more direct and able to divide the mandible, but the disruption of orbicularis ores and philtral architecture exposes the patient to incompetence of the lips after surgery and inappropriate scarring [6]. There are new adjustments in the lateral lip-split techniques which have been introduced which tries to preserve a muscular continuity and aesthetical units of the upper lip [7]. In the present series, both methods had similar oncologic and reconstructive feasibility with regard to equivalent rates of free-flap success. This is in line with literature that indicates that there is no negative effect of incision-type on surgical access or rate of microvascular anastomosis [8]. In oral cavity, however, wound dehiscence and salivary leakage rates were significantly lower with lateral lip-splitting and corroborated by previous studies in which the lateral cuts relax the tension in the wound and preserve the vascularity [9]. Functional result shows a very clear bias favouring the lateral lip splitting approach. Retention of oral competence is also a key factor determining postoperative quality-of-life, including speech, swallowing and social interaction. Quality-of-life instruments that are validated have been used to establish the same findings with respect to better lip function and satisfaction in aesthetics when maintaining philtral subunits [10]. The improvement in speech intelligibility for the lateral group probably occurs as a result of less scar contracture and improved muscle positioning [11]. Aesthetic result is becoming an increasingly important endpoint in head and neck reconstruction. Individuals who survive advanced cancer represent a highly motivated population predominantly interested in facial esthetic rehabilitation that makes flap design particularly relevant [12]. Better aesthetic satisfaction with lateral lip-split incisions is consistent with results seen in modern reconstructive series that stress the importance of preserving subunits [13]. From a surgical point of view, the direct exposure by involving the midline is likely to lead to slightly shorter operative time in case of central lip-split incisions. However, the small difference in dwell time between CR- and PP-BD did not lead to improved clinical outcome and may be offset by increased postoperative morbidity. Enhanced recovery regimes and shortened inpatient stay in lateral lip-split group are further arguments to adopt the procedure in contemporary surgical considerations [14]. There are several limitations to this study, such as the fact that it was performed at a single center with a moderate number of cases. Further follow-up is needed to determine the occurrence of late scarring evolution and mandibular function. Prospective and multicenter randomization trials in the future might generate stronger level of evidence to determine incision placement [15]. In conclusion, the present study indicates that a lateral lip-split incision is not only a functioning-preserving modification of standard mandibulotomy approaches but also does not alter the oncologic safety.

## Conclusion:

Central and lateral lip-split mandibulotomy both offer good access for tumor resection and reconstruction. Lateral lip-split approach provides superior functional and aesthetic results without compromising oncologic safety. Lateral incision design may be considered in the eventthat long-term oral competence and pleasing facial aesthetic are paramount.

## Advancement to knowledge:

This study provides prospective comparative clinical evidence (2020-2026 contexts) demonstrating that lateral lip-split mandibulotomy significantly reduces wound complications, salivary leakage, hospital stay and long-term functional morbidity while maintaining comparable oncologic access and free-flap success rates when compared to central lip-split access, thereby supporting incision design modification as a determinant of postoperative quality-of-life outcomes
